# Exosome-transferred LINC01559 promotes the progression of gastric cancer via PI3K/AKT signaling pathway

**DOI:** 10.1038/s41419-020-02810-5

**Published:** 2020-09-07

**Authors:** Liyan Wang, Xiaotong Bo, Xiaoyuan Yi, Xuhua Xiao, Qinghua Zheng, Lei Ma, Bin Li

**Affiliations:** Gastroenterology Department, Affiliated Hospital of Guilin Medical University, Guilin, Guangxi 541001 China

**Keywords:** Gastric cancer, Biomarkers

## Abstract

Increasing evidence indicates that long non-coding RNAs (lncRNAs) are associated with the progression of human cancers. However, the expression level and function of LINC01559 (long intergenic non-protein coding RNA 1559) in gastric cancer (GC) are rarely reported. Here we found that LINC01559 was upregulated in GC tissues based on GEPIA (Gene Expression Profiling Interactive Analysis) and TCGA (The Cancer Genome Atlas) databases. Also, LINC01559 was expressed at a lower level in GC cells than in mesenchymal stem cells (MSCs). In vitro experiments revealed that silencing LINC01559 remarkably hindered GC cell proliferation, migration and stemness. Then, we identified that LINC01559 was transmitted form MSCs to GC cells via the exosomes. Immunofluorescence staining and electron microscope validated the existence of exosomes in GC cells. Mechanistically, LINC01559 sponged miR-1343-3p to upregulate PGK1 (phosphoglycerate kinase 1), therefore activating PI3K/AKT pathway. Moreover, LINC01559 recruited EZH2 (enhancer of zeste 2 polycomb repressive complex 2 subunit) to PTEN (phosphatase and tensin homolog) promoter, inducing the methylation of PTEN promoter and finally resulting in PTEN repression. Of note, LINC01559 targeted both PGK1 and PTEN to promote GC progression by activating PI3K/AKT pathway. Taken together, our study demonstrated that LINC01559 accelerated GC progression via upregulating PGK1 and downregulating PTEN to trigger phosphatidylinositol 3-kinase/AKT serine/threonine kinase (PI3K/AKT) pathway, indicating LINC01559 as a potential biomarker for GC treatment.

## Introduction

Gastric cancer (GC) is a well-known gastrointestinal tumor with high morbidity around the world^1^. Many factors are responsible for triggering GC such as heavy drinking, irregular dieting, unscientific dieting habit and genetic inheritance^2,3^. Traditional treatments for patients with GC include chemotherapy and surgery. In the latest years, more and more attention has been paid to molecular targeted therapy. However, the types and efficacy of targeted drugs for GC are limited^4^. Thus, it is necessary to find out new and efficient biomarkers for GC treatment.

A great number of long non-coding RNAs (lncRNAs) have been reported to regulate the development of GC by exerting crucial functions in various cellular functions, including cell proliferation, migration, and stemness^5,6^. For example, lncRNA MEG3 suppressed cell proliferation and migration in GC through p53 signaling pathway^7^. THOR enhanced the stemness of GC cells via elevating the stability of SOX9 mRNA^8^. SATB2 was introduced as an inhibitor in GC to repress cell proliferation and migration^9^.

LINC01559 was identified as a biomarker in renal cell carcinoma by a previous study^10^. More importantly, LINC01559 was upregulated in GC tissues based on Gene Expression Profiling Interactive Analysis (GEPIA) and The Cancer Genome Atlas (TCGA) databases. Mesenchymal stem cells (MSCs) could support tumor cell growth. The interaction between MSCs and GC cells performs a crucial role in GC^11^. We identified that, compared with the MSCs, LINC01559 expression was downregulated in GC cells. Thus, present study was designed to investigate on such phenomenon.

Exosomes are defined as small bubbles with sizes around 40–100 nm in diameter, primarily originating from multi-vesicular bodies formed by intracellular lysosomal micro-particles in various types of cells^12^. Exosomes work as crucial factors in cancer communication by the transferring of proteins and RNAs between cells^13^. For example, ZFAS1, transferred by exosomes, accelerated the development of GC^14^. Exosomes-delivered UCA1 led to enhanced tamoxifen resistance in breast cancer^15^. Recently, MSCs-derived exosomes have gradually attracted our attention^16,17^. Especially, exosomes could transmit lncRNAs to cancer cells and therefore accelerate the progression of multiple cancers^18,19^. In the present study, we discovered that LINC01559 was upregulated in GC tissues and could be transmitted from MSCs to GC cells by exosomes.

The phosphatidylinositol 3-kinase/AKT serine/threonine kinase (PI3K/AKT) signaling pathway is generally activated in various types of cancers, including GC. For instance, AK023391 was associated with the occurrence of GC via activating PI3K/AKT signaling pathway^20^. NES1/KLK10 contributed to trastuzumab resistance in GC through stimulating PI3K/AKT signaling pathway. PRL-3 accelerated peritoneal metastasis in GC by PI3K/AKT signaling pathway^21^. In this study, we discovered that LINC01559 could trigger PI3K/AKT signaling pathway via modulating the down-stream targets.

It has been widely accepted that competing endogenous RNA (ceRNA) system has regulatory functions in cancer development. Emerging studies have illustrated that lncRNAs sponge miRNAs to liberate mRNAs so as to cause oncogenic or anti-oncogenic outcomes in cancers including GC. For instance, lncRNA CRAL sponged miR-505 to elevate CYLD expression, thus reversing cisplatin resistance in GC cells^22^. COL1A1-014 served as an endogenous sponge for miR-1273h-5p to upregulate CXCL12^23^. LncRNA MYOSLID sponged miR-29c-3p to antagonize the inhibition of miR-29c-3p on MCL-1 in GC^24^.

In a word, the current study explored whether and how MSCs-derived exosomal LINC01559 affected GC development. Moreover, the role of LINC01559 in ceRNA system and how LINC01559 affected PI3K/AKT pathway in GC were also investigated.

## Materials and methods

### Tissue samples

The ethical approval for this study was acquired from the Ethics Committee of Affiliated Hospital of Guilin Medical University, and the written informed consents were signed by all patients. Total of 80 pairs GC tissue samples were collected between May 2014 and June 2019. Patients received radiotherapy or chemotherapy before surgery were excluded. Tissue samples were snap-frozen after surgical resection in liquid nitrogen and then reserved at −80 °C.

### Isolation of primary MSCs

The fresh tumor tissues were prepared and washed in antibiotics, and then cut into pieces of 1-mm^3^ size and placed to culture dishes for 30 min. Tissue explants were subsequently floated in L-Dulbecco’s Modified Eagle Medium (L-DMEM; Invitrogen, Carlsbad, CA) with 1% Pen/Strep solution and 15% fetal bovine serum (FBS; Gibco, Rockville, MD) in 5% CO_2_ at 37 °C. Tissue pieces were discarded after fibroblast-like cells reached sub-confluence, adherent cells were digested before the passage into culture flasks. At about four passages, the homogeneous cell population (primary MSCs) was acquired for further study.

### Cell lines and reagent

Five human GC cell lines (MKN74, NCI-N87, MKN-45, HGC-27, and AGS) from the American Type Culture Collection (ATCC; Manassas, VA) were cultivated in the DMEM with 1% Pen/Strep solution and 10% FBS in 5% CO_2_ at 37 °C. Insulin like growth factor 1 (IGF-1; 100 ng/mL), the PI3K/AKT pathway activator, was procured from Sigma-Aldrich (St. Louis, MO) to treat HGC-27 and AGS cells.

### Isolation and purification of exosomes

The exosomes secreted by the cultured primary MSCs were isolated by use of the ExoQuick™ solution (System Biosciences; Palo Alto, CA) as per the standard method provided by supplier. The isolated exosome pellets were treated with bicinchoninic acid (BCA) protein assay kit (Beyotime, Shanghai, China) to analyze the protein content in exosome suspension.

### Transmission electron microscopy (TEM)

After treating with 4% paraformaldehyde for fixing, exosome suspension was subjected to the Transmission Electron Microscope grid (Alliance Biosystems, Osaka, Japan). Then, exosome samples were observed via H-7650 transmission electron microscope (Hitachi, Tokyo, Japan).

### Exosome labeling and tracking

As suggested by a previous research^25^, exosome samples were labeled with 1 μM of PKH67 dye (Sigma-Aldrich) for 24 h in light of the user manual to observe the uptake of exosomes in GC cells. 4′,6-diamidino-2-phenylindole (DAPI) solution from Beyotime was added for the staining of cell nuclei. Samples in the slides were fluorescently observed with laser scanning microscope (Carl Zeiss Meditec, Jena, Germany). The uptake of labeled MSCs-derived exosomes by the recipient GC cells was analyzed by flow cytometric assay.

### Dynamic light scattering analysis (DLS)

The particle size distribution of exosome samples was characterized and quantified by DLS (Zetasizer Nano ZS90).

### RNA extraction and real-time quantitative polymerase chain reaction (RT-qPCR)

Total cellular RNA was extracted by applying the TRIZOL reagent (Invitrogen) and then converted into complementary DNA (cDNA). SYBR Green PCR Master Mix (Takara, Kyoto, Japan) was applied for RT-qPCR. Gene expression was calculated by 2^−ΔΔCT^ method, normalizing to glyceraldehyde-3-phosphate dehydrogenase (GAPDH) or U6.

### Colony formation assay

Cell samples were planted at 8 × 10^2^ cells/well to the 6-well plates. Following 14 days of cell culture at 37 °C, samples were treated with 4% paraformaldehyde and crystal violet solution in succession, and then the colonies with over 50 cells were counted manually.

### Transfection

The short hairpin RNAs (shRNAs) specifically against LINC01559, EZH2, PGK1 and relative control shRNAs (sh-NC), along with pcDNA3.1/LINC01559, pcDNA3.1/PTEN and relative control pcDNA3.1 vectors, all these plasmids were produced by Genepharma (Shanghai, China). The miR-1343-3p mimics and NC mimics were also from Genepharma. Cell transfection with indicated plasmids was conducted for 48 h with Lipofectamine 2000 (Invitrogen).

### EdU (5-ethynyl-2′-deoxyuridine) incorporation assay

Cell samples in the 96-well plates were prepared for EdU assay by use of EdU incorporation assay kit (Ribobio, Guangzhou China). After staining nuclei with DAPI, samples were observed by microscope.

### Transwell migration assay

Cell samples in the serum-free culture medium were seeded to the upper chamber of transwell inserts, while the lower chamber was filled with the complete culture medium. Migrating cells were observed by a microscope after fixing in 4% paraformaldehyde and staining with crystal violet solution.

### Sphere formation assay

Cell samples were plated into the 96-well ultralow attachment plates (Corning Inc., New York, NY) containing sphere medium and cultured for 1 week. Images were captured under microscope.

### Subcellular fractionation

PARIS™ Kit (Ambion, Austin, TX) was acquired for conducting subcellular fractionation assay in GC cell samples, following the user guide. Expression levels of LINC01559, U6 and GAPDH were analyzed by RT-qPCR.

### Fluorescence in situ hybridization (FISH)

The RNA FISH probe specifically designed for LINC01559 was procured from RiboBio (Guangzhou, China) and utilized as per the manual. After DAPI staining, images were captured by microscope.

### RNA immunoprecipitation (RIP)

Based on the user guidebook, RIP assay was performed with the EZ-Magna RIP RNA Binding Protein Immunoprecipitation Kit (Millipore, Bedford, MA) by using specific antibodies. Normal mouse IgG served as the negative control (NC). RNAs in precipitates were subjected to RT-qPCR analysis.

### RNA pull down assay and mass spectrometry

Pierce Magnetic RNA-Protein Pull-Down Kit (Thermo Fisher Scientific, Waltham, MA) was applied for RNA pull down assay. Protein extracts from GC cell samples were mixed with biotinylated RNAs and beads for 1 h. Thereafter, the eluted proteins from Bio-LINC01559 group were examined by mass spectrometry analysis as previously described^26^.

### Dual-luciferase reporter assay

The fragments of full-length LINC01559 or PGK1 3’untranslated region (3′UTR) containing wild-type (WT) or mutated (Mut) miR-1343-3p binding sites were inserted into the pmirGLO Dual-Luciferase Reporter Vector (Promega, Madison, WI), so that LINC01559-WT/Mut and PGK1-WT/Mut reporter vectors were acquired. Then, each of above four kinds of recombinant plasmids was co-transfected with miR-1343-3p mimics or NC mimics for 48 h. Besides, pGL3-vector containing PTEN promoter were co-transfected into cell samples with pcDNA3.1/LINC01559 or the NC vector for transcriptional analysis. Finally, the luciferase activities were monitored by Dual-Luciferase Reporter Assay System (Promega).

### Western blot

Cellular protein extracts were treated with 12% sodium dodecyl sulfate-polyacrylamide gel electrophoresis (SDS-PAGE) gel, and then shifted to polyvinylidene fluoride (PVDF) membranes. Primary antibodies against PGK1, PTEN, p-PI3K, PI3K, p-Akt, Akt, p-mTOR (mechanistic target of rapamycin kinase), mTOR, and the loading control GAPDH, together with the secondary antibodies, were all procured from Abcam (Cambridge, MA).

### Chromatin immunoprecipitation (ChIP) assay

DNA-protein cross-links were first prepared, and then cell lysates were sonicated and processed with specific antibodies and beads. Anti-IgG was seen as the NC. Precipitated samples were analyzed by RT-qPCR.

### Chromatin isolation by RNA purification (ChIRP) assay

ChIRP assay was implemented in HGC-27/MSC-exo and AGS/MSC-exo cells in line with the previous protocol^27^. All probes were synthesized with BiotinTEG at the 3’ end.

### In vivo tumor growth experiment

Twenty male BALB/c nude mice (Shi Laike Company, Shanghai, China) were randomly allocated to four experimental groups. The HGC-27/MSC-exo cells transfected with sh-NC or sh-LINC01559#1, and HGC-27 cells transfected with pcDNA3.1 vector (NC) or pcDNA3.1 vector containing LINC01559, were then xenografted into mice. After that, the volume of tumors in above four groups was measured every 4 days. After injection for 28 days, mice were sacrificed and tumor weights were recorded for analysis. In vivo study was implemented under the approval from Animal Care and Use Committee of Affiliated Hospital of Guilin Medical University.

### Immunohistochemistry (IHC)

Tissue samples from in vivo experiment were maintained in 4% paraformaldehyde and embedded in paraffin. Sections (4-μm thick) were obtained for IHC analysis using anti-Ki-67 or anti-PCNA (proliferating cell nuclear antigen) antibody (Santa Cruz Biotechnology, Dallas, TX).

### Statistical analyses

Results were all exhibited as the mean ± SD (standard deviation) of data from independent bio-triplications. Data analysis in each group was processed by Student’s *T*-test, one-way or two-way analysis of variance (ANOVA) using Prism version 5.0 (GraphPad Software, La Jolla, CA). Two-sided *p*-value threshold was set as 0.05 to be statistically significant.

## Results

### LINC01559 served as an oncogene in GC cells

**Fig. 1:**
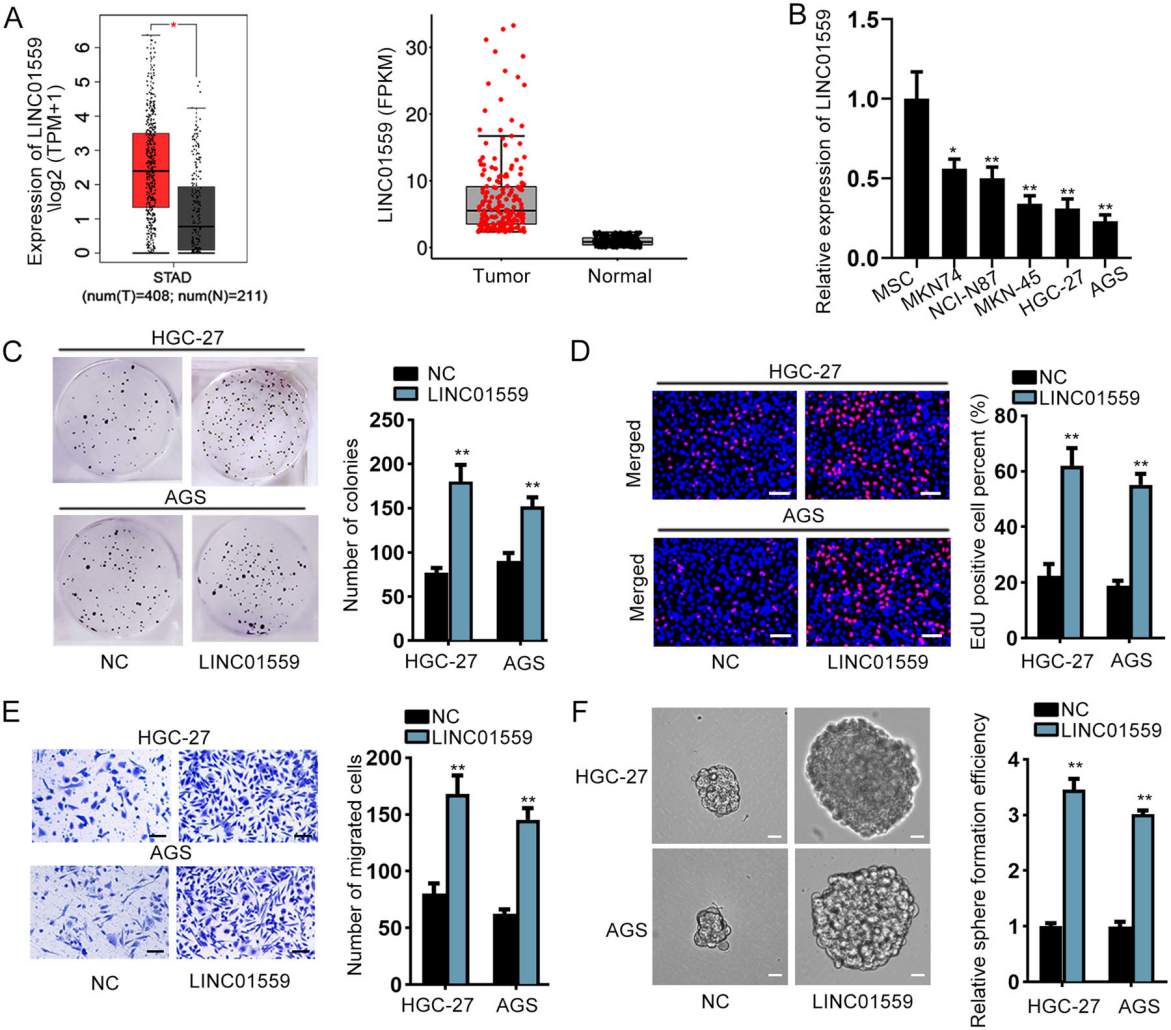
LINC01559 served as an oncogene in GC cells. **a** The expression of LINC01559 in GC tissues based on GEPIA and TCGA databases. Student’s *T*-test. **b** RT-qPCR evaluated LINC01559 expression in GC cells compared to that in MSCs (control cells). One-way ANOVA. **c** Colony formation assay examined GC cell proliferation. Student’s *T*-test. **d** EdU assay (scar bar, 150μm) appraised cell proliferation. Student’s *T*-test. **e** Transwell assay (scar bar, 180μm) assessed cell migration. Student’s *T*-test. **f** Sphere formation (scar bar, 150μm) assay detected the stemness of GC cells. Student’s *T*-test. *P < 0.05, **P < 0.01.

According to both the data from GEPIA (http://gepia.cancer-pku.cn) and TCGA (https://portal.gdc.cancer.gov/), we unveiled that the expression of LINC01559, LINC00483, LINC01296, RAET1E-AS1 and PCED1B-AS1 was significantly high in STAD (stomach adenocarcinoma) tissues in contrast with normal gastric tissues (Fig. 1a, Supplementary Fig. 1A). However, compared to MSCs, only LINC01559 presented a relatively low level in GC cells, while the expression of other 4 lncRNAs exhibited no statistical differences between these two kinds of cells (Fig. 1b, Supplementary Fig. 1B). Due to the fact that HGC-27 and AGS cells had the lowest expression of LINC01559, they were chosen for the next functional assays. Furthermore, we studied the effects of LINC01559 on the function of GC cells. Prior to that, pcDNA3.1/LINC01559 was used to enhance the expression of LINC01559 in these two GC cells (Supplementary Fig. 1C). The outcomes of EdU and colony formation assays revealed that upregulated LINC01559 promoted GC cell proliferation (Fig. 1c, d). Likewise, the migratory ability and sphere formation efficiency were both enhanced by upregulation of LINC01559 (Fig. 1e, f). In summary, LINC01559 was highly expressed in GC tissues and promoted cell proliferation, migration, and stemness.

### MSCs-derived exosomal LINC01559 promoted GC cell proliferation, migration, and stemness

**Fig. 2:**
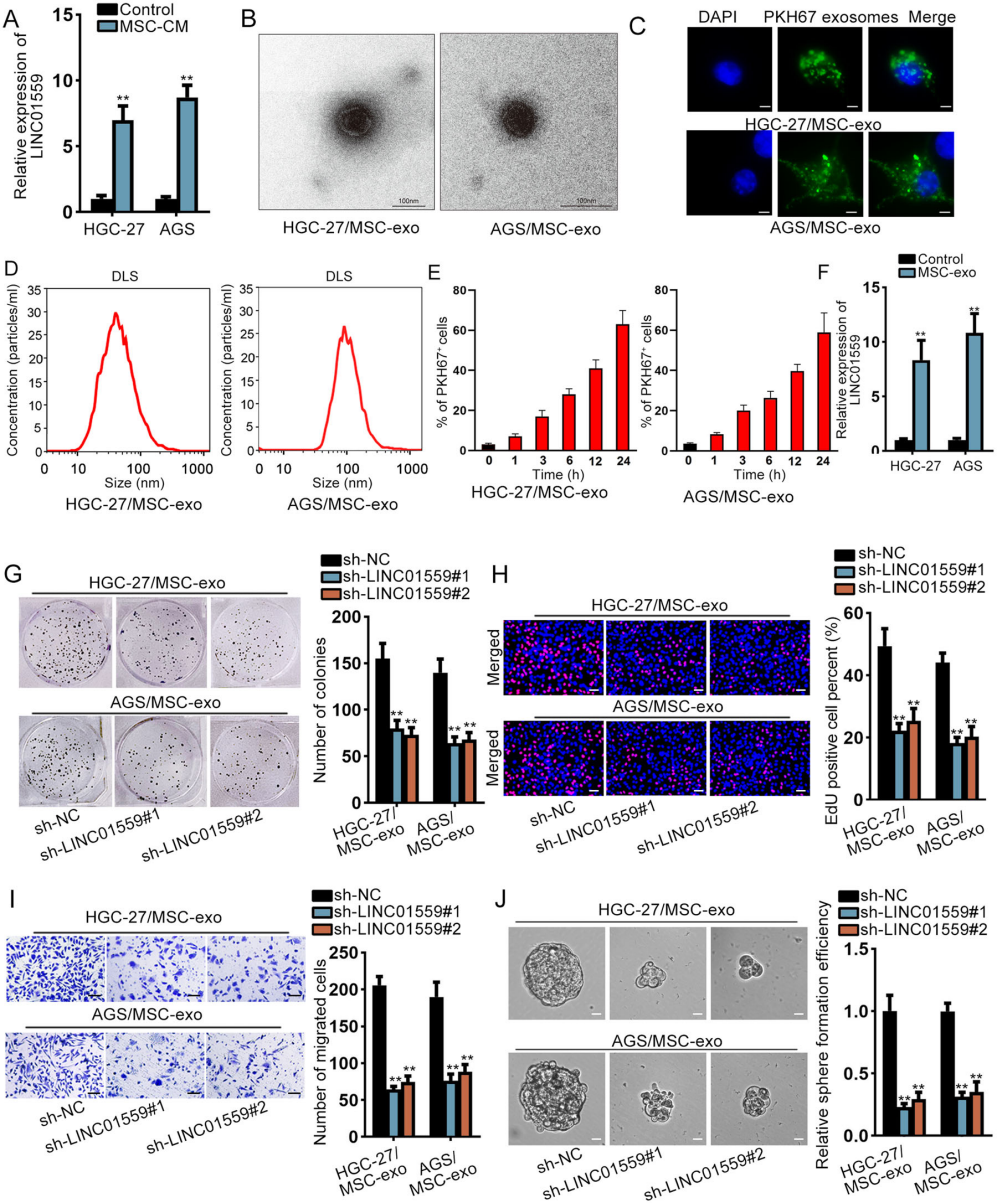
MSCs-derived exosomal LINC01559 promoted GC cell proliferation, migration, and stemness. **a** Expression of LINC01559 in co-cultured GC cells and control GC cells was determined by RT-qPCR. Student’s *T*-test. **b** Representative TEM imaging of exosomes derived from MSCs (scale bar, 100nm). **c** GC cells were cultured by MSCs-derived PKH67-labeled exosomes for 24h. Exosome uptakes by GC cells were demonstrated via a confocal microscope (scale bar, 10μm). GC cells were stained using DAPI (nuclei). **d** Dynamic Light Scattering (DLS) measured the size distribution of exosomes isolated from co-cultured GC cells. **e** Flow cytometric analysis of GC cells after incubation with PKH67-abeled exosomes from MSCs for indicated time. **f** RT-qPCR evaluated LINC01559 expression in GC cells treated with or without exosomes from MSCs. Student’s *T*-test. **g**–**j** Colony formation, EdU (scar bar, 150μm), transwell (scar bar, 180μm) and sphere formation assays (scar bar, 150μm) revealed the impacts of silenced LINC01559 on HGC-27/MSC-exo and AGS/MSC-exo cells in proliferation, migration and stemness. One-way ANOVA. **P < 0.01.

Then, we used MSCs-conditioned medium (CM) to co-culture GC cells and detected the expression of LINC01559, and GC cells without MSC-CM treatment served as the control. As a result, we discovered a higher LINC01559 level in co-cultured GC cells than control cells (Fig. 2a). Thus, we supposed that LINC01559 was transmitted from MSCs to GC cells by exosomes. MSCs from GC patients were dealt with standard methods. We separated exosomes and used transmission electron microscope to validate the identity of exosomes. The relevant results were shown in Fig. 2b. Next, we used PKH67 to mark the exosomes and observed the existence of PKH67-labeled exosomes in HGC-27 and AGS cells (Fig. 2c). Also, the size distribution of exosomes was qualified by DLS analysis (Fig. 2d). Interestingly, GC cells absorbed more and more exosomes from MSCs over time (Fig. 2e). Moreover, we examined the effects of exosomes on the biological behaviors of HGC-27 and AGS cells. As expected, the proliferative abilities of these two cells were increased after exosomes treatment (Supplementary Fig. 1D, E). Meanwhile, exosomes elevated the migratory abilities and sphere formation efficiency of HGC-27 and AGS cells (Supplementary Fig. 1F, G). Besides, LINC01559 was remarkably upregulated in GC cells with the treatment of exosomes (Fig. 2F). After that, sh-LINC01559#1/2 was applied to reduce the expression of LINC01559 in HGC-27 and AGS cells treated with MSC-exo (termed as HGC-27/MSC-exo and AGS/MSC-exo cells subsequently) (Supplementary Fig. 2A). We detected the impacts of silenced LINC01559 on these GC cells. Results exhibited that silenced LINC01559 decreased cell proliferation (Fig. 2g, h), migration (Fig. 2i), and stemness (Fig. 2j). Altogether, exosomal LINC01559 from MSCs could be transferred into GC cells to promote GC cell proliferation, migration and stemness.

### LINC01559 sponged miR-1343-3p to boost the expression of PGK1 in co-cultured GC cells

**Fig. 3:**
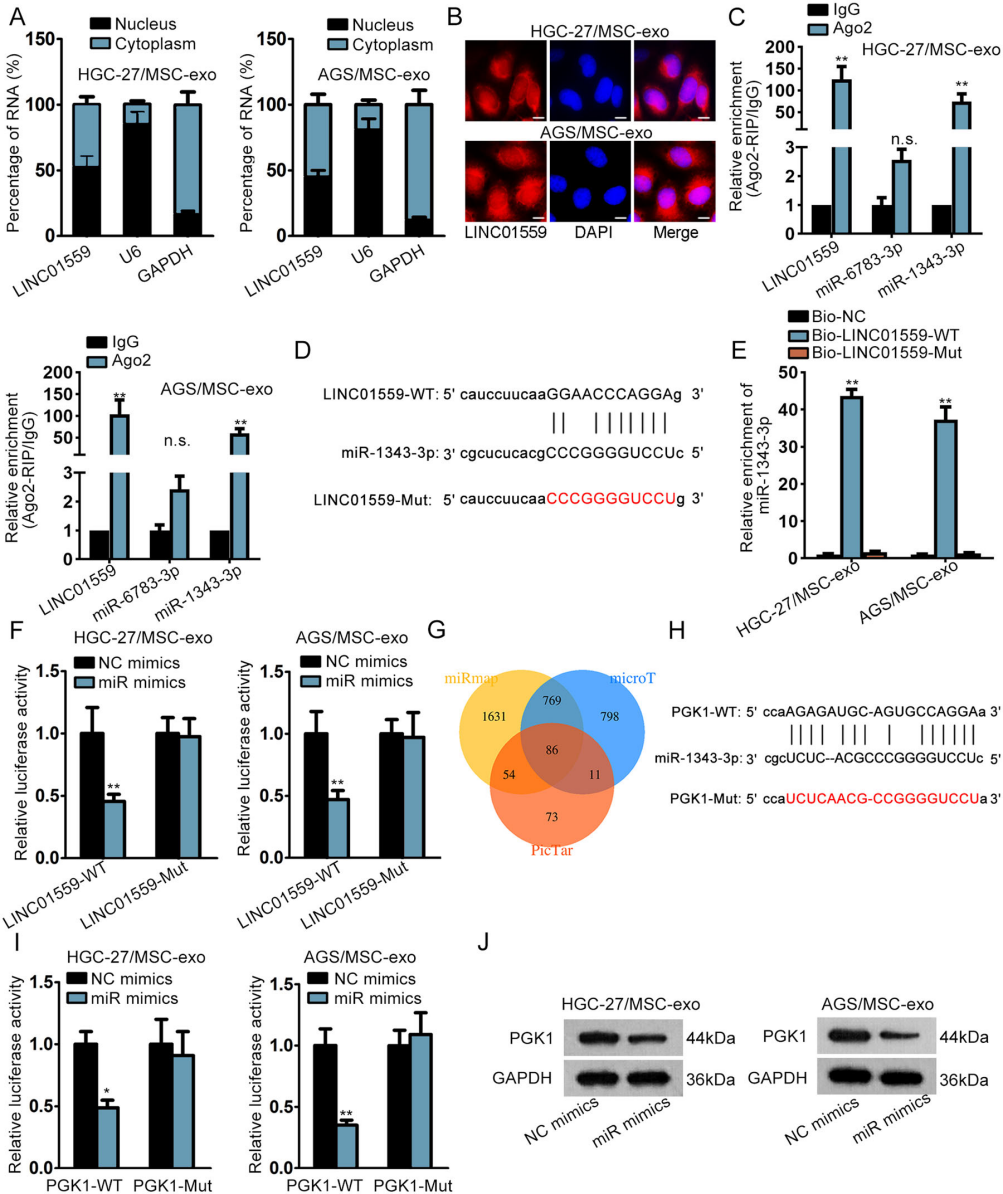
LINC01559 sponged miR-1343-3p to boost the expression of PGK1 in co-cultured GC cells. **a**, **b** Subcellular fractionation and FISH (scar bar, 10μm) were conducted to ascertain subcellular localization of LINC01559. **c** RT-qPCR followed by RIP assays demonstrated the enrichment of LINC01559, miR-6783-3p and miR-1343-3p in Ago2 or IgG group in indicated GC cells. GAPDH served as the normalized control of LINC01559 and U6 acted as that of miR-6783-3p and miR-1343-3p. Student’s *T*-test. **d** The binding sequences between LINC01559 and miR-1343-3p predicted by starBase. **e**, **f** RNA pull down (one-way ANOVA) and luciferase reporter assays (Student’s *T*-test) validated the relationship between LINC01559 and miR-1343-3p. U6 acted as the normalized control of miR-1343-3p enrichment in groups in RNA pull down assay. **g** Venn diagram showed the predicted mRNAs by three diagrams in starBase. **h** The binding sites between miR-1343-3p and PGK1 were predicted by starBase. **i** Luciferase reporter assay validated the relationship between miR-1343-3p and PGK1. Student’s *T*-test. **j** PGK1 protein expression was evaluated by western blot. **P < 0.01. “n.s.” indicates no significance.

After learning about the function of LINC01559 in co-cultured GC cells, we planned to explore the downstream mechanism of LINC01559. The Subcellular fractionation and FISH assays exhibited that LINC01559 was amassed in both nucleus and cytoplasm (Fig. 3a, b). StarBase (http://starbase.sysu.edu.cn) predicted that only miR-6783-3p and miR-1343-3p could bind to LINC01559. Further, data of RIP assays showed that LINC01559 and miR-1343-3p (but not miR-6783-3p) were enriched by Ago2 antibody while no productions were significantly observed in control group (Fig. 3c). The binding sequence between LINC01559 and miR-1343-3p was displayed in Fig. 3d. We mutated the binding sites in LINC01559 to the same sequences of miR-1343-3p, so that miR-1343-3p could not bind to the mutated LINC01559 at all. RNA pull down revealed that miR-1343-3p was noticeably pulled down by Bio-LINC01559-WT rather than Bio-LINC01559-Mut (Fig. 3e). Then, forced expression of miR-1343-3p was verified in cells with miR-1343-3p mimics (Supplementary Fig. 2b). Luciferase reporter assays demonstrated that miR-1343-3p mimics reduced the luciferase activity of reporters with wild type LINC01559 but had no impacts on that of reporters covering mutant LINC01559 (Fig. 3f). Importantly, miR-1343-3p was discovered to have inhibitory influences on the malignant phenotypes of GC cells (Supplementary Fig. 2C–F). Also, miR-1343-3p inhibition partially rescued the effects of LINC01559 silence on colony formation ability of GC cells (Supplementary Fig. 2G). Afterwards, the target genes of miR-1343-3p were searched. From starBase prediction on 3 programs, there were 86 mRNAs which might be targeted by miR-1343-3p (Fig. 3g). Subsequently, we identified PGK1 as the research object since it was the only target gene that was simultaneously upregulated by LINC01559 and downregulated by miR-1343-3p (Supplementary Fig. 2H). The putative binding sites between miR-1343-3p and PGK1, as well as the mutated PGK1 sequences that couldn’t be recognized by miR-1343-3p, were shown in Fig. 3h. Luciferase reporter assays verified the binding between PGK1 and miR-1343-3p at predicted sites (Fig. 3i). Further, we also proved that the protein level of PGK1 was cut down by miR-1343-3p overexpression (Fig. 3J). To summarize, LINC01559 sponged miR-1343-3p to upregulate PGK1 expression in GC.

### LINC01559 accelerated GC progression by activating PI3K/AKT pathway

**Fig. 4:**
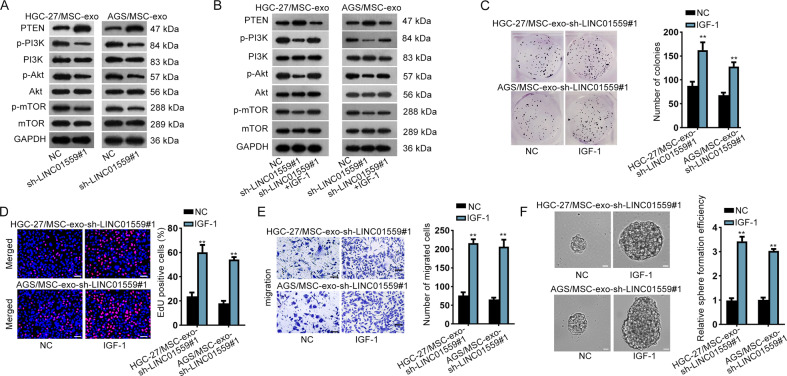
LINC01559 accelerated GC progression by activating PI3K/AKT pathway. **a**, **b** Western blot measured the levels of proteins related to PI3K/AKT pathway in HGC-27/MSC-exo and AGS/MSC-exo cells with LINC01559 depletion or together with IGF-1 treatment. **c**–**f** IGF-1 treatment rescued the effects of LINC01559 inhibition on proliferation, migration and stemness by performing colony formation, EdU (scar bar, 150μm), transwell (scar bar, 180 μm) and sphere formation assays (scar bar, 150 μm). Student’s *T*-test. **P < 0.01.

In this section, the rescue assays were carried out. We firstly examined whether PGK1 was required in LINC01559-regulated GC cellular processes. The results revealed that enhanced expression of PGK1 partially reversed the suppressive effects of depleted LINC01559 on cell proliferation, migration and stemness (Supplementary Fig. 3A–E). A previous study demonstrated that PGK1 activated AKT/mTOR pathway in non-small-cell lung cancer^28^. Hence, we assumed that LINC01559 activated PI3K/AKT pathway via modulating the expression of PGK1. Western blot measured the levels of proteins associated with PI3K/AKT pathway. The data revealed that the protein levels of PGK1, p-PI3K, p-AKT and p-mTOR were decreased while PTEN protein level was increased by depletion of LINC01559 (Fig. 4a). Next, IGF-1, the activator of PI3K^29^, was applied for the rescue assays. As anticipated, IGF-1 treatment obviously reversed LINC01559 deficiency-restrained phosphorylation of PI3K, AKT and mTOR as well as the elevated PTEN level induced by LINC01159 inhibition (Fig. 4b), revealing the re-activation of PI3K/AKT pathway after IGF-1 treatment. Moreover, the results of rescue assays revealed that IGF-1 treatment completely reversed the effects of silenced LINC01559 on cell proliferation, migration and stemness (Fig. 4c–f). Altogether, LINC01559 activated PI3K/AKT pathway to accelerate GC cell proliferation, migration and stemness partially via a PGK1-mediated manner.

### LINC01559 facilitated the methylation of PTEN promoter by recruiting EZH2

**Fig. 5:**
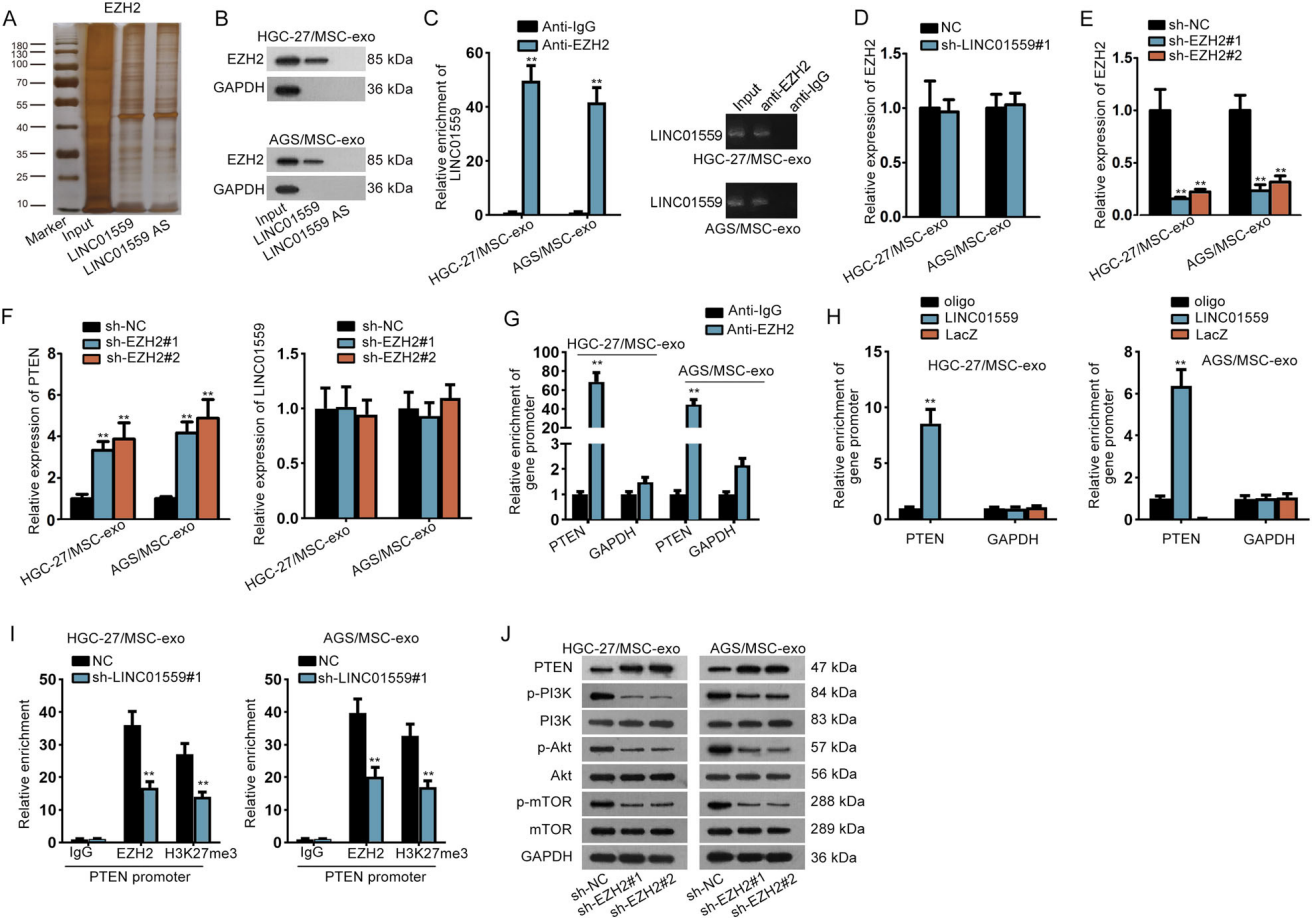
LINC01559 facilitated the methylation of PTEN promoter by recruiting EZH2. **a** RNA-protein pull down and mass spectrometry confirmed that LINC01559 bound to protein EZH2. **b** RNA pull down plus western blot validated the interaction of LINC01559 with EZH2 protein. GAPDH protein was the internal control. **c** RIP assays attested LINC01559 could bind to EZH2, with GAPDH as the normalized control for RT-qPCR analysis of LINC01559 in indicated groups. **d** RT-qPCR examined EZH2 expression in cells with or without silenced LINC01559. Student’s *T*-test. **e** RT-qPCR validated depletion efficiency of EZH2. **f** PTEN or LINC01559 expression was appraised by RT-qPCR. One-way ANOVA. **g** ChIP assay demonstrated PTEN promoter could bind to EZH2, with GAPDH promoter as the negative control. GAPDH was the normalized control for RT-qPCR analysis of PTEN promoter enrichment in indicated groups. **h** ChIRP assay revealed the direct interaction between LINC01559 and PTEN promoter, with GAPDH promoter as the negative control. GAPDH was the normalized control for RT-qPCR analysis of promoter enrichment in indicated groups. One-way ANOVA. **i** ChIP validated that LINC01559 hastened methylation of PTEN by recruiting EZH2. GAPDH was the normalized control for RT-qPCR analysis of PTEN promoter enrichment in indicated groups. Student’s *T*-test. **j** Western blot measured associated proteins of PI3K/AKT pathway by depletion of EZH2. **P < 0.01.

Since the functions of LINC01559 in GC were partially depended on miR-1343-3p-targeted PGK1 but completely mediated by activation of PI3K/AKT pathway, we sought to identify whether LINC01559 regulated another target gene to activate PI3K/AKT pathway independent of miR-1343-3p. LncRNAs are widely reported to bind with specific RBPs and thus have an indirect modulation on target genes. By using RNA-protein pull down and mass spectrometry analysis, we discovered that EZH2 protein could bind to LINC01559 (Fig. 5a, b). In the meantime, RIP assays revealed that LINC01559 could be precipitated by EZH2 (Fig. 5c). Based on above data, together with the finding of a former study that EZH2 could bind to PTEN promoter in GC^30^, we suspected that LINC01559 might affect PTEN transcription in GC via recruiting EZH2. As expected, LINC01559 depletion apparently augmented PTEN expression and had a promotion on the activity of PTEN transcription (Supplementary Fig. 4A–B), without any alterations on EZH2 expression (Fig. 5d). Then, we decreased EZH2 expression via transfecting sh-EZH2#1/2 into cells (Fig. 5e). Interestingly, the outcome of RT-qPCR displayed that inhibiting EZH2 elevated PTEN expression with no influences on LINC01559 level (Fig. 5f), suggesting that EZH2 might affect the transcription of PTEN but not that of LINC01559. Further, ChIP assays demonstrated that EZH2 could bind to PTEN promoter in co-cultured GC cells (Fig. 5g). More intriguingly, we unveiled that LINC01559 could directly interact with PTEN promoter in these two cells (Fig. 5h). Based on these results, we suspected that LINC01559 could recruit EZH2 to PTEN promoter via interacting with EZH2 protein. Such speculation was verified by ChIP assays since that LINC01559 depletion could apparently decline the binding of EZH2 to PTEN promoter, resulting in alleviated tri-methylation of H3K27 at PTEN promoter (Fig. 5i). Besides, the data of western blot revealed that downregulated EZH2 boosted PTEN level and reduced the activity of PI3K/AKT pathway (Fig. 5j). Meanwhile, we proofed that the potentiation of reduced LINC01559 on PTEN expression could be attenuated after EZH2 enhancement (Supplementary Fig. 4C). All in all, LINC01559 hindered PTEN transcription to activate PI3K/AKT signaling by recruiting EZH2.

### LINC01559 affected GC development by targeting PTEN and PGK1

**Fig. 6:**
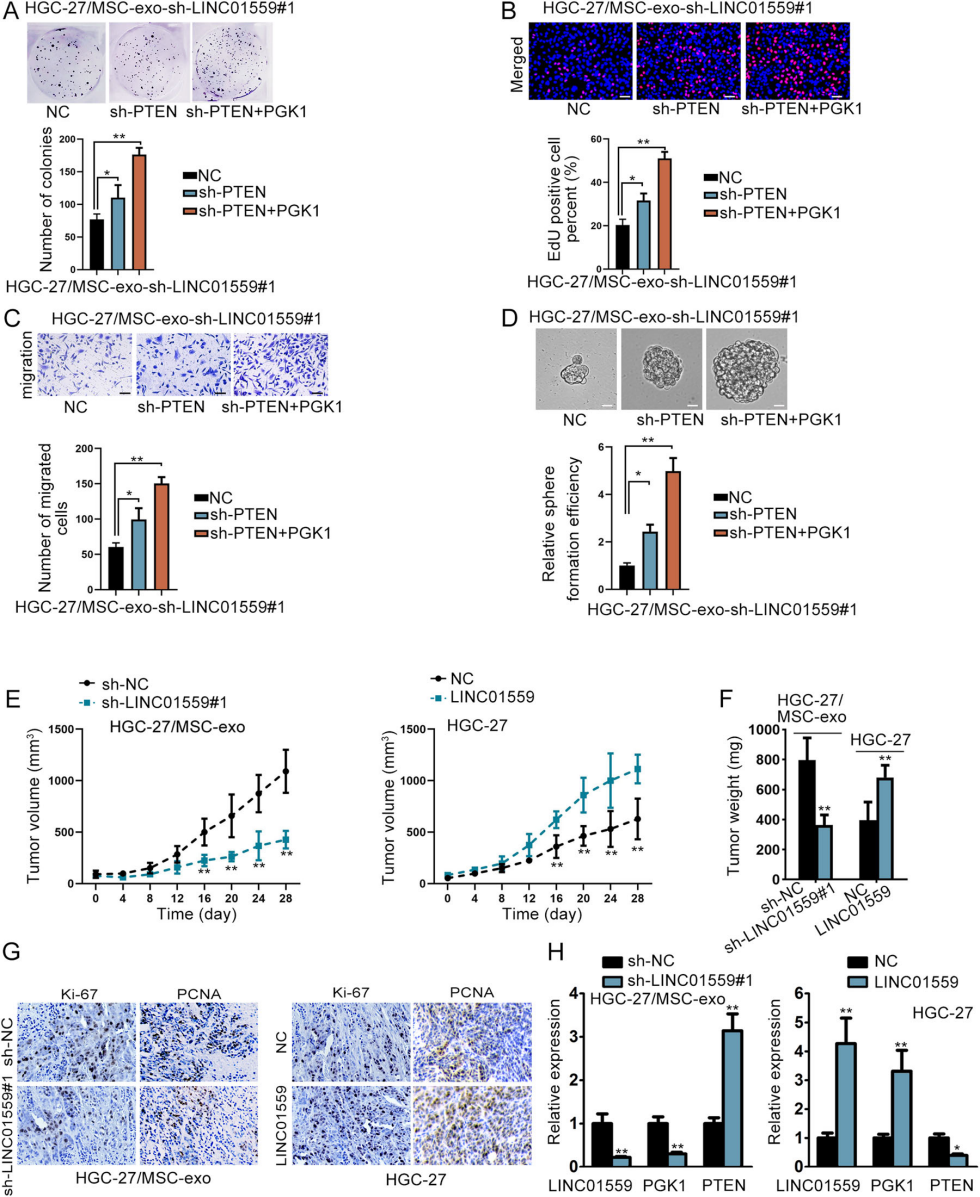
LINC01559 affected GC development by targeting PTEN and PGK1. **a**–**d** The rescue effects of PTEN and PGK1 on the proliferation, migration and stemness in LINC01559-silenced HGC-27/MSC-exo cells were demonstrated by colony formation, EdU (scar bar, 150μm), transwell (scar bar, 180μm) and sphere formation assays (scar bar, 150μm). One-way ANOVA. **e**, **f** Tumor growth curve and tumor weight were shown. For Fig. 6e, the statistical method was two-way ANOVA while for Fig. 6f was Student’s *T*-test. **g** IHC measured Ki-67 and PCNA positivity in indicated tumors. **h** RT-qPCR detected LINC01559, PGK1, and PTEN expression in xenografts from indicated groups. Student’s *T*-test. *P < 0.05, **P < 0.01.

Next, we carried out rescue assays again. Before that, silence efficiency of PTEN was validated (Supplementary Fig. 4D). The results from rescue assays manifested that downregulation of PTEN only partial reversed the impacts of LINC01559 deficiency on cell proliferation, migration and stemness, whereas PTEN suppression combined with PGK1 elevation completely normalized LINC01559 depletion-induced phenomena (Fig. 6a–d). Subsequently, we conducted in vivo experiments to validate the influence of LINC01559 on tumor growth. Thus, MSC-exo treated HGC-27 cells after transfection with sh-NC or sh-LINC01559#1, HGC-27 cells transfected with vector control or pcDNA3.1/LINC01559, these four kinds of HGC-27 cells were separately injected into mice in four different groups. As a result, LINC01559 suppression hampered the growth of tumor while overexpression of LINC01559 had the opposite effects (Fig. 6e, f). Data of IHC displayed that the positivity of Ki-67 and PCNA was reduced under LINC01559 silence but augmented by upregulation of LINC01559 (Fig. 6g). RT-qPCR also exhibited that the levels of LINC01559 and PGK1 were cut down and PTEN expression was elevated in tumors with downregulated LINC01559, whereas Xenografts with LINC01559 overexpression showed the contrary results (Fig. 6h). To sum up, LINC01559 contributed to the malignancy in GC by targeting PGK1 and PTEN.

### Clinical values of LINC01559/miR-1343-3p/PGK1/PTEN axis in GC

**Fig. 7:**
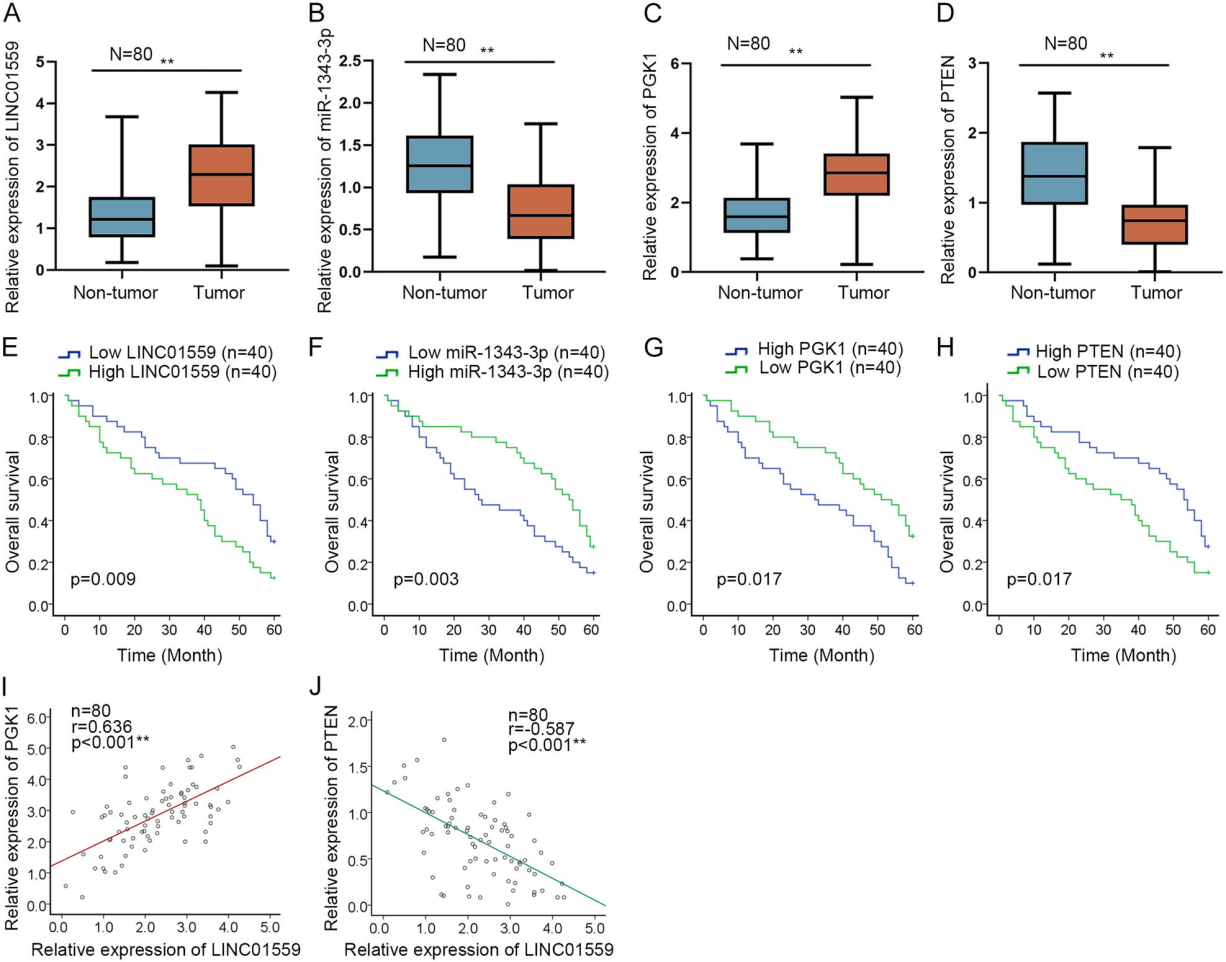
Clinical values of LINC01559/miR-1343-3p/PGK1/PTEN axis in GC. **a**–**d** RT-qPCR estimated the expression of LINC01559, miR-1343-3p, PGK1 and PTEN in 80 pairs of GC tissues. **e**–**h** The prognostic values of LINC01559, miR-1343-3p, PGK1 and PTEN in GC were determined by Kaplan–Meier curves. **i**, **j** Pearson’s correlation analysis uncovered the association of LINC01559 expression with PGK1 or PTEN level in 80 GC tissues. **P < 0.01.

In subsequent step, we estimated whether above findings were clinically applicable. Consistently, we discovered elevated LINC01559 and PGK1 levels as well as repressed miR-1343-3p and PTEN levels in GC tissues compared to matched non-cancerous ones (Fig. 7a, d). More significantly, it exhibited that higher expression of LINC01559 or PGK1 in GC patients tended to suffer a worse prognosis, while those with higher miR-1343-3p or PTEN levels were likely to have a better survival rate (Fig. 7e–h). Then, an evident positive correlation between PGK1 and LINC01559 in expression in above GC tissues was verified by Pearson’s correlation analysis (Fig. 7i), while such phenomenon was also observed in TCGA data (Supplementary Fig. 4E). In contrast, we disclosed that PTEN expression tended to be negatively associated with LINC01559 level in clinical samples (Fig. 7j), although we didn’t find more evidence for this in TCGA database (Supplementary Fig. 4F). Thus, we believed that LINC01559 might have a meaningful value in GC development through its regulation on PGK1 and PTEN.

**Fig. 8:**
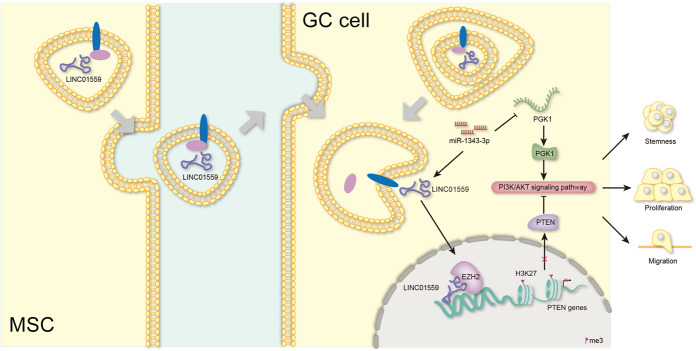
Graphical abstract. Graphical diagram of LINC01559/miR-1343-3p/PGK1/PTEN axis in facilitating GC cell proliferation, migration and stemness.

Based on all the findings, we concluded that LINC01559, transmitted via exosomes from MSCs to GC cells, could sequester miR-1343-3p to augment PGK1 and recruit EZH2 to epigenetically repress PTEN, ultimately leading to activated PI3K/AKT pathway to aggravate GC cell proliferation, migration and stemness (Fig. 8).

## Discussion

Emerging lncRNAs are demonstrated to have efficiencies in regulating GC development. Our study focused on the role of LINC01559 in GC. At the beginning, LINC01559 was discovered to be upregulated in GC tissues. The functional assays certified that LINC01559 could fortify proliferative and migratory abilities as well as stemness characteristic in GC cells. All these data implied that LINC01559 elicited an oncogenic function in GC. Then, we identified that LINC01559 expression was relatively low in GC cells compared with that in MSCs. Intriguingly, when we co-cultured GC cells with MSC-CM, LINC01559 was upregulated in such GC cells.

Recently, exosomes have attracted more and more attention in cancer. Li and his team argued that exosomes-delivered SOX2OT facilitated epithelial-mesenchymal transition (EMT) and stemness in pancreatic ductal adenocarcinoma^31^. Exosomal miR-27a derived from GC cells modulated the transformation of fibroblasts into carcinomas-related fibroblasts^32^. In our study, we found that exosomes derived from MSCs could be received by GC cells. Further, LINC01559 could be transmitted from MSCs to GC cells by exosomes. More importantly, the exosomal LINC01559 promoted GC progression via enhancing GC cell proliferation, migration, and stemness.

Numerous studies have advocated that ceRNA regulatory system plays a significant part in cancers, including GC. For instance, LINC01133 suppressed GC progression via sponging miR-106a-3p to modulate APC and Wnt/β-catenin pathway^33^. LINC01234 promoted the progression of GC by modulating CBFB and sponging miR-204-5p^34^. In present study, LINC01559 was indicated to amass both in nucleus and cytoplasm. In this regard, the potential ceRNA mechanism involving LINC01559 in GC was explored. Subsequently, miR-1343-3p was verified to be sponged by LINC01559. Zhou revealed that miR-1343-3p negatively regulated TEAD4to work as an inhibitor in gastric tumorigenesis^35^. Present study also uncovered that miR-1343-3p served as a tumor suppressor in GC. Besides, miR-1343-3p inhibition partially rescued the effects of suppressed LINC01559 on GC cell proliferation. After that, PGK1 was verified as the downstream target of miR-1343-3p. PGK1 expression was decreased by enhanced miR-1343-3p and was increased by upregulated LINC01559. PGK1 is a famous oncogene^36,37^ and is previously reported to activate PI3K/AKT pathway^38^.

PI3K/AKT pathway is a common-sighted pathway associated with the occurrence and development of various cancers. Activation of this pathway is featured by phosphorylated AKT. Some cases revealed that PI3K/AKT pathway exerted important functions in GC. XLOC_006753 strengthened drug resistance of GC cells by PI3K/AKT pathway^39^. Pectolinarigenin imposed autophagy and apoptosis as well as cell cycle arrest in GC cells via PI3K/AKT pathway^40^. Based on the experimental data, we validated that LINC01559 could trigger the activity of PI3K/AKT pathway via upregulation of PGK1. Moreover, the effects of LINC01559 silence on GC cellular functions were completely attenuated by IGF-1, which was the activator of PI3K/AKT pathway. However, PGK1 partially rescued the such effects. Thus, we assumed that LINC01559 could activate PI3K/AKT signaling via another way independent of miR-1343-3p.

PTEN is a common tumor suppressor and has a negative impact on the activation of PI3K/AKT pathway. Previous research demonstrated that EZH2 could bind to PTEN promoter^30^. It was convinced in our study that LINC01559 could interact with both EZH2 protein and PTEN promoter, which enabled it to recruit EZH2 to PTEN promoter. EZH2 was widely reported to mediate H3K27me3, a transcription-suppressive histone mark in promoter regions. For example, CLDN14 was silenced via EZH2-induced H3K23me3 to serve as a new prognostic biomarker in hepatocellular carcinoma^41^. EZH2 was described to be an underlying therapeutic target for H3K27M-mutant pediatric glioma^42^. Similarly, present study revealed that LINC01559 enhanced tri-methylation of H3K27 at PTEN promoter by recruiting EZH2, resulting in repressed PTEN expression in GC. In summary, all the data in our research indicated that MSCs-derived exosomal LINC01559 could activate PI3K/AKT pathway to hasten the development of GC via sponging miR-1343-3p to upregulate PGK1 and by recruiting EZH2 to repress PTEN. Our work highlighted that LINC01559 might be a promising biomarker for GC treatment in the future.

## Supplementary information

Supplementary figure legend

Supplementary Fig. 1

Supplementary Fig. 2

Supplementary Fig. 3

Supplementary Fig. 4
