# Supplementary material for: Exosome-transferred LINC01559 promotes the progression of gastric cancer via PI3K/AKT signaling pathway
Source: Cell Death Dis. 2020 Sep 7;11(9):723. doi: 10.1038/s41419-020-02810-5 (PMC7477231; doi:10.1038/s41419-020-02810-5)
Supplement: Supplementary file 1 — Supplementary figure legend [file 41419_2020_2810_MOESM1_ESM.docx]

**Supplementary Figure Legends**

**Supplementary Figure 1** (A) GEPIA database revealed the expression of LINC00483, LINC01296, RAET1E-AS1 and PCED1B-AS1 in STAD samples and normal ones. (B) The expression of LINC00483, LINC01296, RAET1E-AS1 and PCED1B-AS1 was detected in GC cell lines and MSCs. One-way ANOVA. (C) RT-qPCR detected overexpression efficiency of LINC01559. Student’s T-test (D-G) Effects of MSCs-derived exosomes on cell proliferation, migration and stemness were demonstrated in colony formation, EdU, transwell and sphere formation assays. Student’s T-test. *P < 0.05, ^**^P < 0.01.

**Supplementary Figure 2** (A-B) LINC01559 expression was detected by RT-qPCR in cells transfected with sh-LINC01559#1/2. For A was One-way ANOVA and for B was Student’s T-test. (C) MiR-1343-3p expression was probed by RT-qPCR in cells transfected with miR-1343-3p mimics. Student’s T-test. (D-F) The function of miR-1343-3p in co-cultured GC cell proliferation, migration and stemness. Student’s T-test. (G) RT-qPCR revealed knockdown efficiency of miR-1343-3p and colony formation assay validated the rescue effects of miR-1343-3p inhibition on LINC01559 depletion in GC cells. For the first data, the statistical method is Student’s T-test while for the second one is one-way ANOVA. (H) Venn diagram revealed that PGK1 was the only mRNA which was up-regulated by LINC01559 and down-regulated by miR-1343-3p at the same time. ^**^P < 0.01.

**Figure Supplement 3** (A) Overexpression efficiency of PGK1 was evaluated by RT-qPCR. Student’s T-test. (B-E) Up-regulation of PGK1 partially rescued silenced LINC01559 on proliferation (scar bar, 150μm), migration (scar bar, 180μm) and stemness (scar bar, 150μm) in rescue assays. One-way ANOVA. ^*^P < 0.05, ^**^P < 0.01.

**Figure Supplement 4** (A) PTEN expression in cells with or without silenced LINC01559 was appraised by RT-qPCR. Student’s T-test. (B) Luciferase reporter assay demonstrated that LINC01559 decreased the luciferase activity of PTEN promoter. Student’s T-test. (C) RT-qPCR revealed the expression of PTEN in cells with LINC01559 silence or together with EZH2 overexpression. One-way ANOVA. (D) Depletion efficiency of PTEN was validated via RT-qPCR. One-way ANOVA. (E-F) TCGA data showed the correlation of LINC01559 with PGK1 or PTEN in STAD tissues. ^**^P < 0.01.
