# Supplementary figures and images for: Exosome-transferred LINC01559 promotes the progression of gastric cancer via PI3K/AKT signaling pathway
Source: Cell Death Dis. 2020 Sep 7;11(9):723. doi: 10.1038/s41419-020-02810-5 (PMC7477231; doi:10.1038/s41419-020-02810-5)

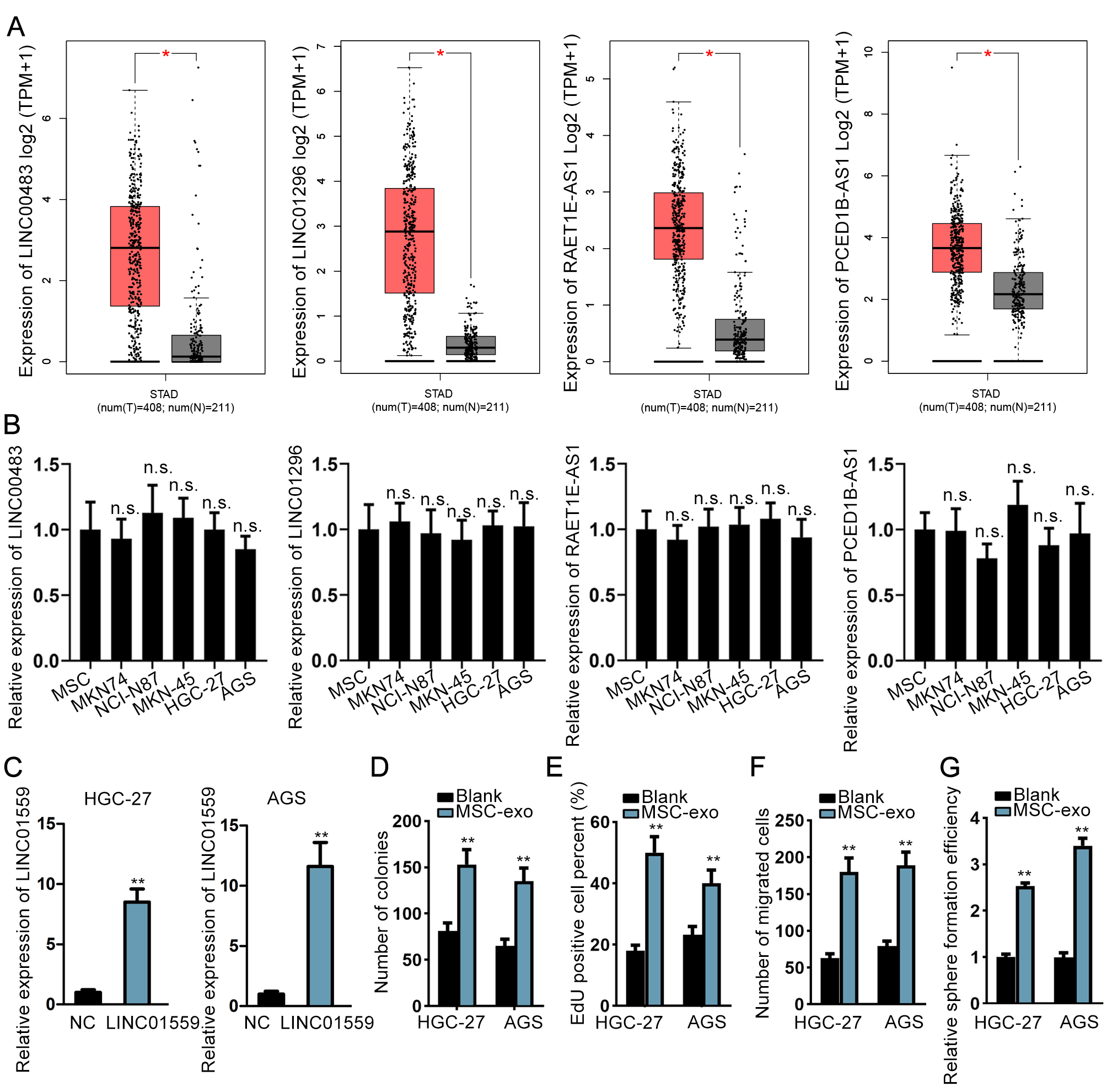

Supplement: Supplementary file 2 — Supplementary Fig. 1 [file 41419_2020_2810_MOESM2_ESM.tif]

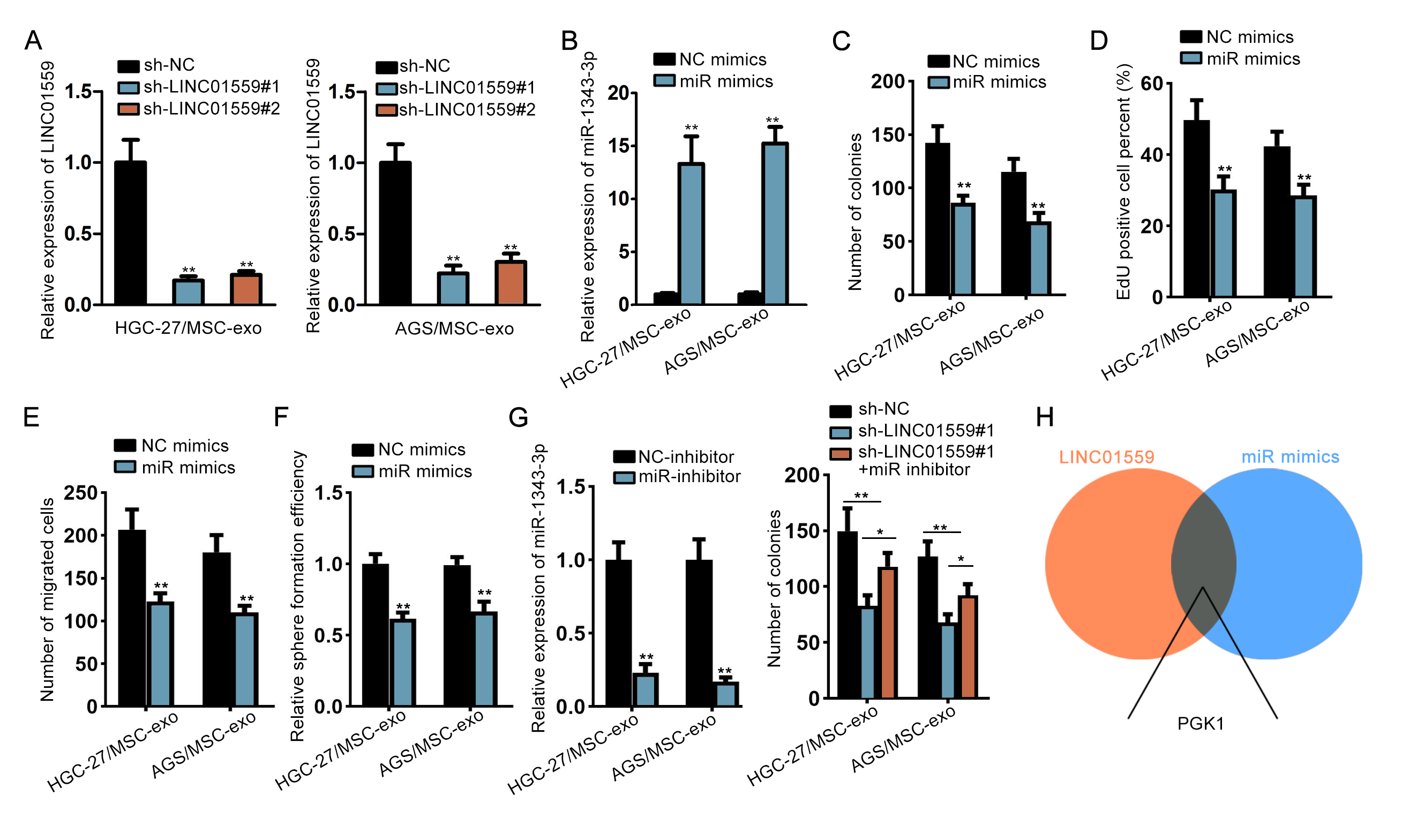

Supplement: Supplementary file 3 — Supplementary Fig. 2 [file 41419_2020_2810_MOESM3_ESM.tif]

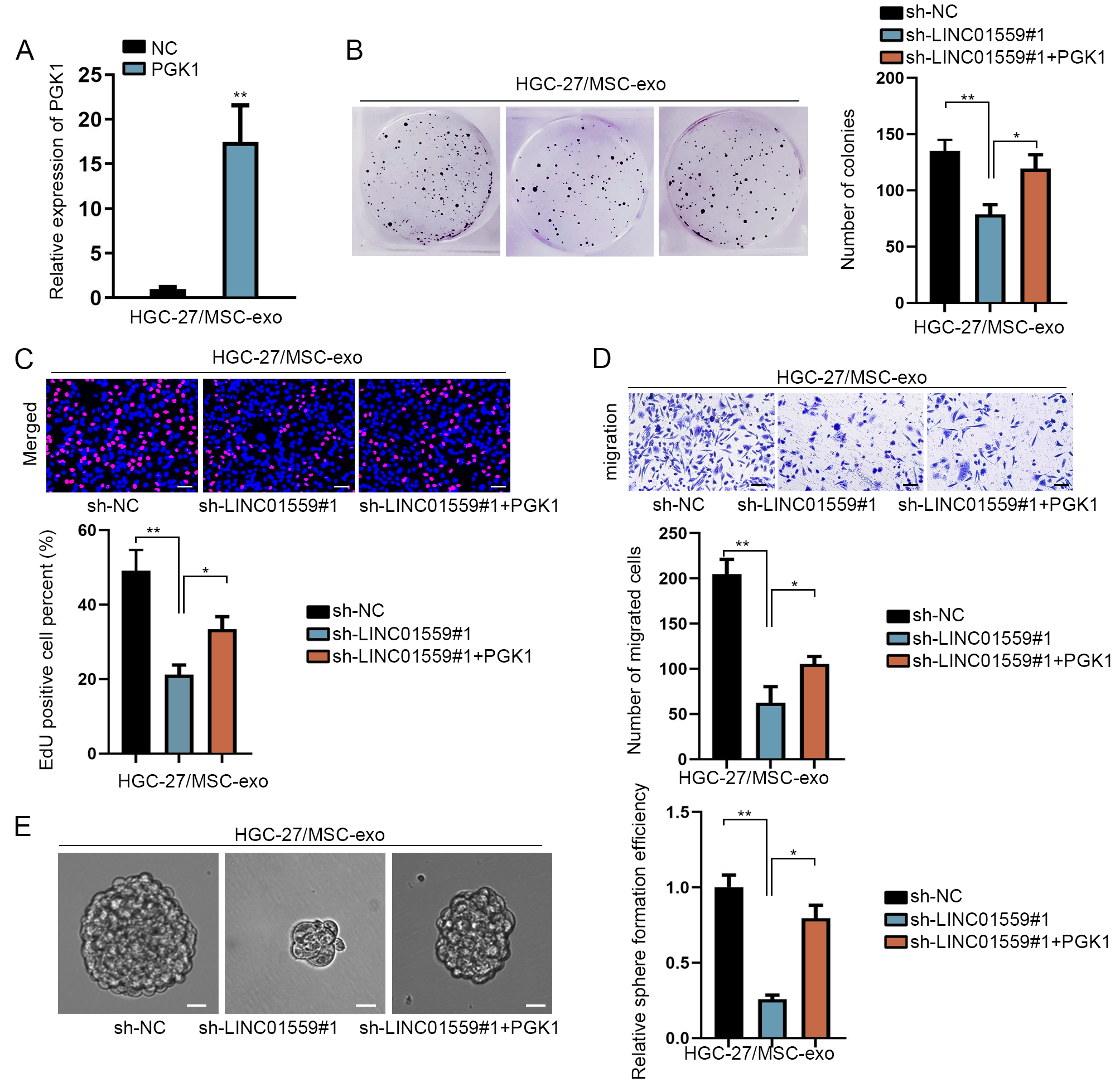

Supplement: Supplementary file 4 — Supplementary Fig. 3 [file 41419_2020_2810_MOESM4_ESM.tif]

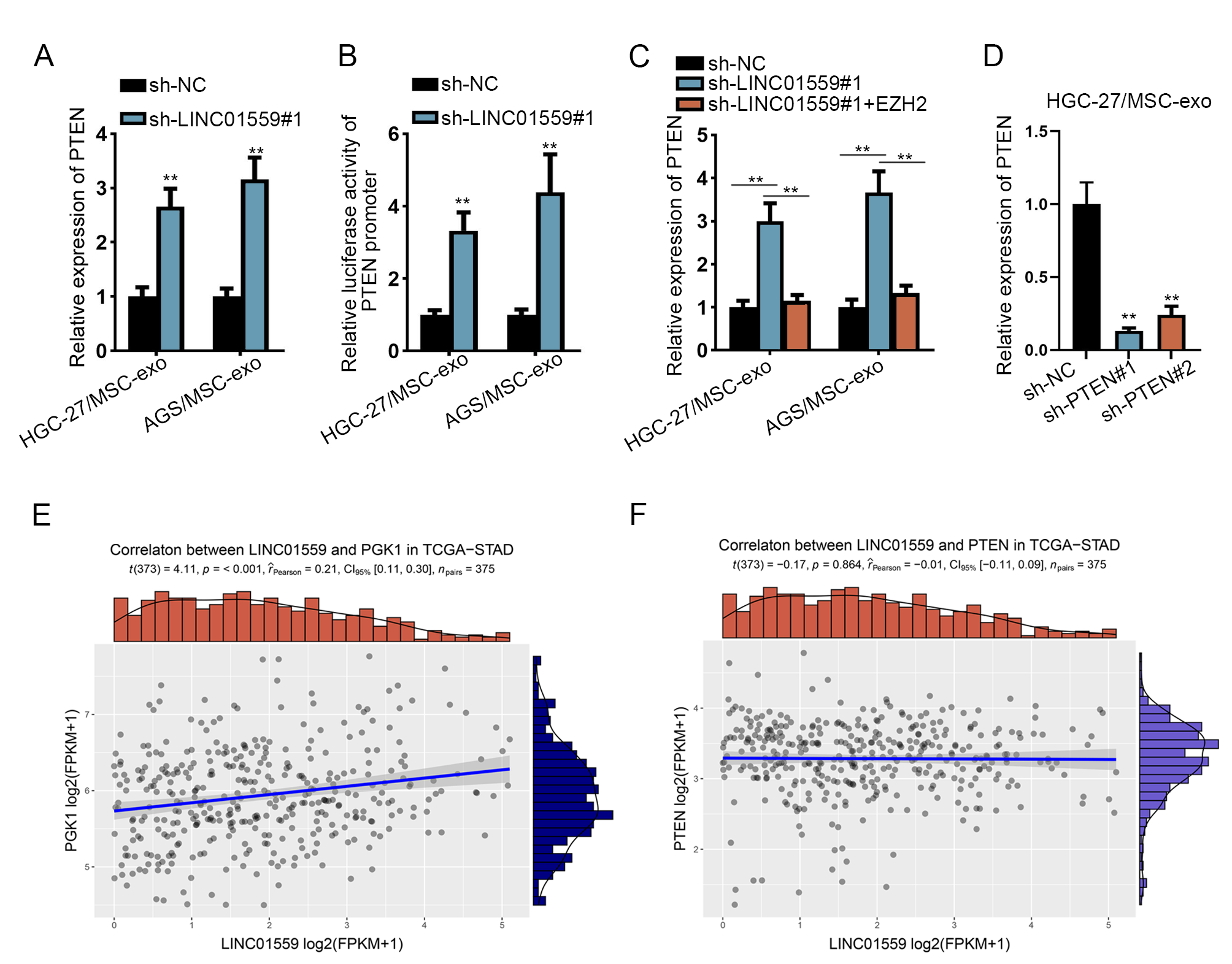

Supplement: Supplementary file 5 — Supplementary Fig. 4 [file 41419_2020_2810_MOESM5_ESM.tif]
